# Sick leave in the prodromal phase of multiple sclerosis and its association with diagnostic delay: A short report

**DOI:** 10.1177/20552173241305982

**Published:** 2024-12-18

**Authors:** Alejandra Machado, Emma Pettersson, Kristina Alexanderson, Jan Hillert, Emilie Friberg

**Affiliations:** Division of Insurance Medicine, Department of Clinical Neuroscience, 27106 Karolinska Institutet, Stockholm, Sweden; Division of Neurology, Department of Clinical Neuroscience, 27106 Karolinska Institutet, Stockholm, Sweden; Division of Insurance Medicine, Department of Clinical Neuroscience, 27106 Karolinska Institutet, Stockholm, Sweden

**Keywords:** Multiple sclerosis, epidemiology

## Abstract

The prodromal features of multiple sclerosis (MS) are non-specific and are prevalent in the general population. Several studies indicate an increased use of healthcare resources by individuals with MS in the years preceding their diagnosis, suggesting a trend of deteriorating health prior to the clinical manifestation of MS. This study aimed to capture the possible associations of sick leave with the timing of the diagnosis of MS. Our findings suggest that sick leave with neurological diagnoses - excluding MS, and other diagnoses during the year before MS onset is associated with a shorter time to MS diagnosis.

## Introduction

The prodromal phase of multiple sclerosis (MS) is associated with a wide and varying range of symptoms and with higher healthcare usage prior to the clinical onset of MS,^1^ even when compared to references.^2^ Such healthcare usage results from a number of different disorders, e.g., fatigue, pain, migraine, anemia, bowel and bladder issues, sleep disturbances, depression or mood disorders, dermatological-related issues, and low cognitive performance.^1,3,4^ These general symptoms are difficult to associate with the MS diagnosis itself, and may therefore mislead towards other diagnosis, delaying the accurate diagnosis of MS.^5^ Additionally, other conditions that mimic MS symptoms can contribute to this diagnostic delay, particularly in patients with varied neurological symptoms.^6^ Further, higher numbers of days of sick leave have been observed among people in the years before MS diagnosis than among population-based references without MS diagnosis.^7,8^

With the growing interest in prodromal symptoms of MS, our goal was to explore whether information on sick leave can provide valuable insights as a proxy of prodromal MS features. These subtle signs and symptoms may appear in an early latent period (prodromal phase) years before the debut of the typical clinical symptoms of MS manifest (MS onset), and which later enable the diagnosis of MS.^9^ To our knowledge, there are no published studies identifying whether being on sick leave prior to MS onset can be associated with timing of the diagnosis of MS. Therefore, we aimed to investigate whether sick-leave diagnoses one year prior to MS onset may impact the identification of the underlying MS diagnosis.

## Materials and methods

A longitudinal population-based cohort study was conducted including all MS patients listed in the Swedish MS Register (SMSreg) with an onset date in the period 2006–2020, who were later diagnosed with MS at the age of 18 or older.

### Data sources

Individual-level data was linked using the unique personal identity numbers issued to all residents in Sweden, from three nationwide registers: SMSreg (age at onset, dates of MS onset and of MS diagnosis), the Micro Data for Analysis of the Social Insurance (MiDAS; for sick-leave (SL) spells (dates, diagnoses)) and the Longitudinal integrated database for health insurance and labour market studies (LISA; for other demographic information corresponding to the year of onset: sex, educational level, born or not in Sweden, civil status, children living at home, and type of living area based on degree of urbanization of municipality of residence).

### Study population

The final cohort included 7037 MS patients, with available information on both onset and diagnosis dates, and who in the year preceding MS onset date, lived in Sweden and had no disability pension.

### Sick leave measures

Sick-leave spells of more than 14 continuous days within the 365 days prior to MS onset date were noted, irrespective of diagnosis, and categorized as indication of all-cause SL (Yes = 1/No = 0). Additionally, these SL spells were classified using ICD-10 codes, allowing indication of diagnosis-specific SL, though not exclusive, for the following diagnoses:

- Mental (F00-F99)
- Other neurological (G00-G99, excluding MS diagnosis (G35))
- Musculoskeletal (M00-M99)
- Cardiovascular (I00-I99)
- Cancer (C00-D48)
- Injury (S00-T98)
- Eye & vision (H00-H59)
- Respiratory (J00-J99)
- Dermatological (L00-L99)
- Genitourinary (N00-N99)
- Other (all remaining ICD-10 codes) including missing diagnoses (0.3% of mean net SL days).

### Statistical analyses

The distribution of demographic variables for all patients and by all-cause SL indication is presented in Table 1. The absolute difference of SL days was explored with the Mann-Whitney U test. Cox proportional hazards analysis was performed to estimate the hazard ratio (HR) of receiving an MS diagnosis (event), using the diagnostic delay (time between onset and diagnosis of MS in days, as dated in the SMSreg) as the time-to-event. Since all patients eventually receive an MS diagnosis, the results of the survival analysis focus on the time until diagnosis, indicating either a shorter or longer diagnostic delay. Predictor variables (all-cause SL or diagnosis-specific SL) were analyzed in separate models. Covariates included in the survival models were year of onset and sex (see caption in Table 2 for further details).

**Table 1.** Distribution for demographic and clinical variables by all-cause sick leave (SL) (yes/no) and in total.

	SL = yes (n = 1055)	SL = no (n = 5982)	Total (N = 7037)
	n(%)	n(%)	n(%)
**Age at onset**			
<20 years	<20 (0.4%)	273 (4.6%)	277 (3.9%)
20–29 years	244 (23.1%)	1938 (32.4%)	2182 (31.0%)
30–39 years	348 (33.0%)	1815 (30.3%)	2163 (30.7%)
40–49 years	292 (27.7%)	1268 (21.1%)	1560 (22.2%)
≥50 years	167 (15.8%)	688 (11.5%)	855 (12.2%)
*Mean (Sd)*	*35.0 (11.0)*	*38.5 (10.4)*	*35.5 (11.0)*
*Median [IQR]*	*38* *[30–46]*	*33* *[26–43]*	*34* *[27–43]*
**Sex**			
women	784 (74.3%)	3983 (66.6%)	4767 (67.7%)
men	271 (25.7%)	1999 (33.4%)	2270 (32.3%)
**Educational level**			
non-university	735 (69.7%)	3309 (55.3%)	4044 (57.5%)
university	1320 (30.3%)	2673 (44.7%)	2993 (42.5%)
**Born in Sweden**			
yes	928 (88.0%)	5179 (86.6)	6107 (86.8%)
no	127 (12.0%)	803 (13.4%)	930 (13.2%)
**Married**			
No	638 (60.5%)	4049 (67.7%)	4687 (66.6%)
Yes	417 (39.5%)	1933 (32.3%)	2350 (33.4%)
**Children <18 living at home**			
yes	472 (44.7%)	2252 (37.6%)	4313 (61.3%)
no	583 (55.3%)	3730 (62.4%)	2724 (38.7%)
**Type of living area**			
large city	394 (37.3%)	2572 (43.0%)	2988 (42.1%)
medium-sized city	432 (40.9%)	2321 (38.8%)	2753 (39.1%)
rural areas	229 (21.7%)	1089 (18.2%)	1318 (18.7%)

For ethical reasons, small frequency counts for individuals are reported as “<20”.

Abbreviations: SL, Sick leave; Sd, standard deviation, IQR, Interquartile Rate.

**Table 2.** Hazard ratios (HR) with 95% confidence intervals (CI) for delay of MS diagnosis, using all-cause sick leave (SL) and diagnosis-specific SL in the 365 days before MS onset, among 7037 MS patients.

	N = 7037	Unadjusted models		Adjusted models	
	n(%)	HR	[95% CI]	HR	[95% CI]
All-cause sick leave (SL)	1055(15%)	1.119	[1.048–1.194]	1.139	[1.066–1.216]
Diagnosis-specific (SL)	n (%)	HR	[95% CI]	HR	[95% CI]
SL - mental	244 (3.5%)	1.133	[0.997–1.287]	1.035	[0.910–1.176]
SL - other neurological (non-MS)	96 (1.4%)	**1**.**448**	**[1.184–1.771]**	**1**.**338**	**[1.094–1.637]**
SL - musculoskeletal	229 (3.3%)	0.954	[0.837–1.089]	0.982	[0.861–1.121]
SL - cardiovascular	<20 (0.2%)	1.035	[0.643–1.666]	1.355	[0.842–2.182]
SL - cancer	21 (0.3%)	0.936	[0.610–1.437]	1.144	[0.745–1.756]
SL - injury	63 (0.9%)	0.998	[0.803–1.239]	0.926	[0.746–1.150]
SL - eye & vision	67 (1.0%)	1.042	[0.819–1.326]	1.239	[0.973–1.577]
SL - respiratory	31 (0.5%)	**0**.**624**	**[0.438–0.888]**	0.746	[0.524–1.062]
SL - dermatological	<20 (0.1%)	0.941	[0.489–1.809]	0.885	[0.460–1.702]
SL - genitourinary	<20 (0.2%)	0.972	[0.552–1.713]	1.235	[0.701–2.177]
SL - other	262 (3.7%)	1.086	[0.960–1.228]	**1**.**176**	**[1.039–1.331]**

*Reference*: Indication of sick leave (SL) diagnosis (No).

Adjusted models were controlled for sex and year of MS onset.

For ethical reasons, small frequency counts for individuals with a specific SA diagnosis are reported as “<20”. To note: preliminary analysis were conducted to examine demographic factors, including age at onset, sex, educational level, country of birth (Sweden or elsewhere), civil status, presence of children <18 years at home, and type of living area based on the degree of urbanization, in relation to diagnostic delay. The results indicated that only age at onset significantly was associated with time to MS diagnosis. However, age was significantly correlation with the year of onset. Given the study's timespan and variations in diagnostic criteria, we decided to include the year of MS onset instead of the age at onset. Nevertheless, we performed additional sensititivity analysis, adjusting for both age at onset and year of onset: this did not alter the results. Finally, significant sex differences were observed in specific sick-leave diagnoses, with women having more SL, particularly due to mental diagnoses, genitourinary diagnoses, and “other conditions” (data not shown). Therefore, sex was also considered as a covariate in the analysis.

## Results

In the cohort, 68% of the participants were women and the average age at MS onset was 35.5 years (Table 1). Additionally, 15% had at least one sick-leave spell in the 365 days prior to MS onset (Table 2). The average diagnostic delay was 502 days (16 months; median = 206 days). Individuals with any SL (n = 1055) had a shorter diagnostic delay (median days, Interquartile Range (IQR): 42–521) compared to those without SL (n = 5982; Median = 215days, IQR: 68–650). This difference was statistically significant (U = 2,803,265; p < 0.001).

Results from the survival models showed that having any SL during the year prior to MS onset was associated with a higher likelihood of receiving the MS diagnosis sooner, suggesting a shorter time to diagnosis (or a shorter diagnostic delay) compared to those with no SL (Table 2). This also applied for other non-MS neurological SL diagnoses (i.e., neurological diagnosis excluding MS). In contrast, those with SL with respiratory diagnosis had a longer diagnostic delay. When adjusted for sex and year of MS onset, individuals who had any SL before MS onset still had a shorter diagnostic delay (HR = 1.139; 95% CI[1.066–1.216]). Furthermore, SL due to other non-MS neurological diagnoses (excluding MS) and “other” diagnoses category were also associated with a shorter diagnostic delay (HR = 1.338; 95% CI[1.094–1.637] and HR = 1.176; 95% CI[1.039–1.331], respectively). Within the category “other diagnoses”, the highest percentage of these SL spells (1.3% of the total sample) were due to “Symptoms, signs and abnormal clinical and laboratory findings, not elsewhere classified” (ICD-10 code: R00–99), followed by “Pregnancy, childbirth and the puerperium” (codes O00–99; 0.9%) (data not shown in Table 2).

## Discussion

While the diagnostic delay for MS has decreased over the past years, owing to advancements in medical technology and improved diagnostic criteria, a significant time gap between the onset of clinical symptoms and confirmation of the disease still exists.

Our findings indicate that individuals with MS who had any sick leave in the year before onset had, on average, a shorter diagnostic delay compared to those without SL during the same period. However, a considerable variability in the diagnostic delay within the SL group was present, probably owing to the wide range of symptom presentations.^5^ After adjusting for year of onset and sex, individuals with any SL prior to MS onset were diagnosed with MS more quickly that those without SL. Specifically, shorter diagnostic delays were observed when SL was given for non-MS neurological diagnoses or “other” diagnosis, such as other symptoms and laboratory findings not elsewhere classified.

Our findings suggest that earlier engagement with healthcare, according to SL information, and particularly for non-MS neurological SL diagnoses, can imply quicker confirmation of MS. This finding is not surprising, given the overlap between MS symptoms and those of other neurological disorders,^10^ but also reflects the common misdiagnosis of conditions that mimic MS, particularly in patients with varied neurological symptoms.^6^ Nevertheless, ascertainment bias may play a role in our findings and the interaction between various neurological conditions. Therefore, suggesting that the diagnostic process for MS might enhance vigilance, thereby accelerating the detection of other conditions rather than delaying them, as is commonly assumed.^5^ These findings highlight the potential utility of sick-leave information during the prodromal phase of MS. Monitoring sick-leave patterns, particularly those of neurological conditions, may enable healthcare providers to better identify individuals with MS, leading to earlier diagnosis and facilitating interventions.

This study benefits from the use of high-quality register data, though it has some limitations. First, information on SL spells shorter than 15 days was not available. Second, we only had information about the first main SL diagnosis, and did not capture comorbidities (secondary diagnoses).

Despite being exploratory, these findings hold significant clinical relevance, suggesting that information about SL during the prodromal phases of diseases, such as MS, could raise awareness and provide additional insights that may facilitate timely diagnosis. Further studies are warranted to confirm these findings, particularly to determine if the presence of SL related to other non-MS neurological diagnoses, could act as a risk indicator for a MS diagnosis compared to matched references. Analyzing these patterns may lead to earlier diagnosis and improve prognoses, ultimately benefiting the patient's life course.
